# Selection of Peptide Mimics of HIV-1 Epitope Recognized by Neutralizing Antibody VRC01

**DOI:** 10.1371/journal.pone.0120847

**Published:** 2015-03-18

**Authors:** Anton N. Chikaev, Anastasiya Yu. Bakulina, Ryan C. Burdick, Larisa I. Karpenko, Vinay K. Pathak, Alexander A. Ilyichev

**Affiliations:** 1 State Research Center of Virology and Biotechnology VECTOR, Koltsovo, Novosibirsk region, 630559, Russia; 2 HIV Drug Resistance Program, National Cancer Institute-Frederick, Viral Mutation Section, Frederick, Maryland, 21702, United States of America; New York State Dept. Health, UNITED STATES

## Abstract

The ability to induce anti-HIV-1 antibodies that can neutralize a broad spectrum of viral isolates from different subtypes seems to be a key requirement for development of an effective HIV-1 vaccine. The epitopes recognized by the most potent broadly neutralizing antibodies that have been characterized are largely discontinuous. Mimetics of such conformational epitopes could be potentially used as components of a synthetic immunogen that can elicit neutralizing antibodies. Here we used phage display technology to identify peptide motifs that mimic the epitope recognized by monoclonal antibody VRC01, which is able to neutralize up to 91% of circulating primary isolates. Three rounds of biopanning were performed against 2 different phage peptide libraries for this purpose. The binding specificity of selected phage clones to monoclonal antibody VRC01 was estimated using dot blot analysis. The putative peptide mimics exposed on the surface of selected phages were analyzed for conformational and linear homology to the surface of HIV-1 gp120 fragment using computational analysis. Corresponding peptides were synthesized and checked for their ability to interfere with neutralization activity of VRC01 in a competitive inhibition assay. One of the most common peptides selected from 12-mer phage library was found to partially mimic a CD4-binding loop fragment, whereas none of the circular C7C-mer peptides was able to mimic any HIV-1 domains. However, peptides identified from both the 12-mer and C7C-mer peptide libraries showed rescue of HIV-1 infectivity in the competitive inhibition assay. The identification of epitope mimics may lead to novel immunogens capable of inducing broadly reactive neutralizing antibodies.

## Introduction

Design of a safe and effective HIV-1 vaccine is extremely important in view of world-wide spread of the AIDS epidemic. High HIV-1 genetic variability, early establishment of the latent virus reservoir, its ability to escape adaptive immunity, and the absence of distinct immune correlates of protection are all factors that present real challenges for vaccine development. Understanding the mechanisms underlying the origin and antigenic specificity of antibodies that efficiently neutralize a wide range of HIV-1 subtypes is crucial to developing a successful HIV-1 vaccine. Antibodies capable of neutralizing a broad spectrum of HIV-1 isolates were recently found in sera of a small number of HIV-infected individuals [1]. The discovery of these broadly neutralizing antibodies (bNAbs) has provided an enormous impetus to the HIV vaccine research [2]. If the vaccine could prime the immune system to produce these broadly neutralizing antibodies before exposure to HIV, they could potentially prevent infection. The current goal for AIDS vaccine researchers is to try to engineer vaccine immunogens that can coax the body’s immune system to make potent, broadly neutralizing antibodies against HIV.

In recent years, a few dozen broadly neutralizing antibodies have been isolated and characterized [3, 4]. A group of bNAbs that recognize the conserved CD4-binding site (CD4-BS) of glycoprotein gp120 of HIV-1 (Env) are of interest because they are able to block virus binding to CD4 cell receptor, resulting in prevention of virus penetration into cells. VRC family of bNAbs are particularly noteworthy because they can neutralize an extraordinarily wide range (up to 90%) of circulating HIV-1 isolates [5].

Development of HIV-1 vaccine candidates that are capable of inducing anti-CD4-BS nAbs is greatly complicated by the fact that these antibodies bind only to native viral Env trimer. Expression of a combination of several conformational epitopes in a synthetic antigen seems to be a rather complicated and challenging task so far in modern molecular biology. There are several approaches based on the use of different forms of Env as an immunogen. One approach to vaccine design is to create soluble, recombinant antigenic mimics of the functional Env trimers that mimic the native form of Env found on the virion surface [6–8]. Another strategy concerns the development of immunogens based on a monomer Env structure. Such proteins have been derived from stabilized core Env proteins, whose surface can be modified with the use of targeted mutations resulting in imitation of the CD4-BS [5, 9, 10]. It should be noted, however, that such CD4-BS immunogens may contain some undesirable epitopes in addition to the desirable one. It is likely that these undesirable epitopes will be immunodominant, resulting in a weak antibody response against the target epitope that cannot efficiently neutralize viral infectivity.

Another approach for developing HIV-1 vaccine employs scaffolds based on informatics and epitope transplantation. It was shown that the structure derived by transplantation of the epitope recognized by bNAb 2F5 into acceptor scaffold was able to induce immune response in guinea pigs very similar to antibody 2F5. It has also been demonstrated that the monoclonal antibodies elicited by epitope-scaffold constructs replicate 2F5 structure-specific recognition of the gp41 MPER [11]. The limitation of this method is that only the linear motifs could be used for transplantation into scaffold; thus, it is not applicable in the case of discontinuous epitopes.

The difficulties mentioned above could be evaded by using artificial antigens that contain peptide mimics selected using combinatorial biology methods such as phage display [12–18], Signature Motif Method (Immunosignaturing) [19], and computational prediction of neutralizing epitopes targeted by bNAbs [20]. This approach eliminates the undesirable epitopes in the immunogen structure. Phage peptide libraries contain peptides of certain length and with a random amino-acid composition that are exposed on the surface of filamentous phage as part of the minor coat protein. Libraries are constructed in order to express a foreign peptide at the N-terminus of bacteriophage surface coat protein that is located on the surface of virion and is accessible for interaction with a protein ligand (or antibody) used for affinity selection [21]. Phage libraries have a large diversity of approximately 10^9^ independent peptides which permits detection of sequences that are able to mimic the conformation of the native epitope through biopanning [21].

The aim of this work was to identify phage clones from the phage libraries exposing peptides that specifically bind to bNAb VRC01 and to assess the ability of selected peptides to mimic the HIV-1 epitope recognized by VRC01.

## Methods

### Phage library and biopanning

Phage peptide libraries (New England BioLabs) displaying random cyclic 7-mer and linear 12-mer peptides at the N-terminus of the coat protein, pIII, were panned with bNAb VRC01 (obtained from the NIH AIDS Research and Reference Reagent Program). The procedure for screening the phage display library was performed according to the manufacturer’s instructions. Briefly, ELISA plates were coated with 2 μg/well of MAb VRC01 overnight at 4°C. Wells were blocked for 2 h with 1% bovine serum albumin (BSA) (Sigma, USA) in TBS and incubated with an aliquot of the 12-mer phage library (2×10^11^ phage particles) in binding buffer (TBS, 0.1% Tween-20 or 0.5% Tween-20). Unbound phage particles were removed by extensive washing with TBS/0.5% Tween-20. Bound phages were eluted with 0.2 M Glycine—HCL/0.1% BSA, pH 2.2 and immediately neutralized. Phages were amplified in Escherichia coli ER2738, precipitated from the bacterial culture supernatant with polyethylene glycol, and subjected to titer determination before the next round.

A total of three successive rounds of panning were performed with both of the libraries. After the third positive selection, the titers of the phages were determined, single clones were picked, and then single-stranded DNA from individually isolated clones was purified according to the manufacturer’s instructions and sequenced. Sequencing was performed by the Center of collective using “Genomics” Institute of Chemical Biology & Fundamental Medicine SB RAS (Novosibirsk, Russia). Sequences encoding the phage peptides were generated, edited, translated and analyzed using the BioEdit software.

### Dot-blot assay

Bacteriophages were applied on nitrocellulose membrane at concentrations of 5×10^10^ plaque-forming units (pfu), 1×10^10^ pfu, and 0.2×10^10^ pfu (in 2 μl of PBS). After drying, the membrane was blocked with 3% BSA for 1 h at room temperature, and then incubated for 2 h at 37°C with the solution of primary antibodies of 1 mg/ml concentration. Washing of unbound antibodies was carried out three times for 10 min with 0.05% PBST (1X PBS + 0.05% Tween-20, pH 7.4). Goat antibodies against human IgG (Thermo Scientific Pierce Antibodies, USA) conjugated with alkaline phosphatase were used as secondary antibodies. The nitrocellulose membrane was incubated with conjugate diluted 1:5000 in blocking buffer for 1 h. Unbound components were washed three times for 10 min with 0.05% PBST. BCIP/NBT was used as a chromogenic substrate.

### Peptide synthesis and Neutralization Competition Assay

Displayed peptides were synthesized by GenScript (Piscataway, NJ, USA) with >80% purity and tested for their ability to compete with HIV-1 for binding to MAb VRC01. We developed a high-throughput 96-well plate HIV-1 infectivity assay to assess the neutralization capacity of bNAbs. Wild-type virus (NL4–3), which was preincubated with different dilutions of VRC01 for 30 min at 37°C, was used to infect TZM-bl indicator cells that express the luciferase reporter protein upon infection. Using this assay, we determined the concentration of VRC01 needed to inhibit HIV-1 infection by 90% (6.26 μg/ml). Two dilutions of competitor peptides (stock concentration of ~1 mg/ml and its 5-fold dilution) and 6.26 μg/ml of IgG VRC01 (corresponds to the measured IC90 value) were preincubated for 30 min at 37°C, and then this mixture was added (1:1 by volume) to HIV-1, and the resulting mixture was incubated for an additional hour at 37°C. The mixture of peptides, VRC01, and HIV-1 was then added (1:1 by volume) to the TZM-bl indicator cells. The luciferase activity was measured 48- to 72-h post-infection using a luciferase substrate (Promega). To check for specificity, some peptides were tested for their ability to interfere with neutralization by IgG1 b12 [22]. First, we determined the concentration of IgG1 b12 needed to inhibit HIV-1 infection by 90% (12.5 μg/ml) and then determined the ability of the peptide to interfere with neutralization as described above using the stock concentration of peptide.

### Modelling Software

To compare peptide sequences and gp120 structure we used Pepitope server [23] and our in-house software pdMap. All gp120 regions aligned to the peptides by Pepitope server were outside of the known VRC01 epitope. pdMap software has similar algorithm, but searching of similarity was restricted by user defined area of a protein. Model of peptide-antibody complex was built in PyMOL software.

## Results

### Biopanning and screening

Ph.D-12 and Ph.D-C7C libraries displaying random linear 12-mer and cyclic 7-mer peptides at the N-terminus of the coat protein, pIII, were screened with MAb VRC01. As the first indicator of successful biopanning, an increase of phage titers after each round of panning was observed. The titer of eluted phages after each round of panning increased from 9.5×10^5^ pfu/ml (first round) to 4.2×10^6^ pfu/ml (second round), and finally to 1.6×10^9^ pfu/ml (third round) in experiments with Ph.D-12. Corresponding titers after Ph.D-C7C library biopanning were 5.6×10^5^, 1.3×10^6^, and 2.7×10^9^ pfu/ml. A total of 90 phage plaques and 58 phage plaques for the 12-mer and cyclic 7-mer libraries, respectively, were randomly chosen for colony screening after three successive rounds of biopanning.

The ssDNAs from these phage plaques were sequenced. The peptide sequences were derived using BioEdit and aligned with MimoDB online tool. In the case of complete match of peptides from independent phage clones we further tested only one of these clones. A number of repeated peptides are shown in Table 1. Some of the selected phage clones carried peptides that were identified as non-specific (plastic-binding peptides). Those peptides were excluded from the following experiments (data not shown).

**Table 1:** Identification of 12-mer and cyclic 7-mer peptides that bind to bNAb VRC01 by panning Ph.D-12 and Ph.D-C7C phage peptide libraries.

Phage library	Peptide number	Intensity of spots at different titers^a^			Peptide sequence	no. phage clones
		5×10^10^	5×10^9^	1×10^9^		
**Ph.D-12**	E1	++++	+++	+	VSWPELYKWTWS	16
	E2	++++	+++	+	ITAPELYAWFGS	1
	E3	++++	+++	+	LTWGEMHTWTVQ	20
	E4	+++	+++	-	LTMEELTRWSVY	1
	E5	++	+	-	ITLPELHAWKEN	1
	E6	++++	+++	-	ITIQEITAWPES	1
	E7	+++	+	-	LTNQELLTWTAY	1
	E8	++	++	-	ITNAELTNWNNG	2
	E9	++	+	-	MDLAELSNWPHA	1
	E10	+	-	-	TTFDILDYWTSN	4
	E11	++	-	-	LSIADLYRWNTS	1
	E12	+	-	-	GQPWTTWLESNT	2
	E13	++	+	-	ITTSEIYNWRDT	3
	E14	-	-	-	WQIWEYWPMDHN	1
	E15	+-	+-	-	VTLGELVSWPAE	1
	E16	+	+-	-	LTLEELLFWKSP	1
	E17	++	+	-	LTRLELLEWDSP	1
	E18	+++	++	-	LSWEELLRWASP	1
	E19	+++	++	-	LTHTELLHWNGM	1
	E20	+	-	-	ITQADVWAWDTS	1
	Negative control	-	-	-		
**Ph.D-C7C**	C1	++++	+++	-	CSWTLLGYC	5
	C2	+++	++	-	CIWEFLGFC	1
	C3	++++	+++	-	CNWEFWKYC	5
	C4	++++	++	-	CEWSLWSFC	6
	C5	++	+	-	CTFTYWGFC	1
	C6	+	+-	-	CPWYLMGYC	1
	C7	+	-	-	CNWSLLSFC	1
	C8	-	-	-	CEWTWFGYC	1
	C9	++++	+	-	CSWNLMGFC	5
	C10	+-	-	-	CLWSLTGFC	1
	C11	+++	++	-	CQWTYYNFC	1
	C12	+++	++	+	CEWRYWEYC	1
	C13	-	-	-	CSWSLNGFC	3
	C14	++	+	+-	CPWVLHGFC	4
	C15	++++	++	-	CNWEFWKYC	5
	C16	++	-	-	CDWLLHGFC	1
	C17	++++	+	-	CSWNLMGFC	5
	C18	++	+	-	CPWMLSGFC	1
	C19	+++	-	-	CTWSLSGFC	1
	C20	+	+-	-	CLWMLEKFC	1
	C21	-	-	-	CTHSRAGSC	1
	C22	-	-	-	CSWSLLDFC	1
	C23	++++	+++	++	CTWTLLSFC	4
	Negative control	-	-	-		

^a^ The signal intensities are from dot-blot analyses using different titers of phage-displayed peptides. The numbers of isolated phage clones which contained the corresponding peptide are indicated in the far right column.

### Dot-blot analysis

We carried out dot-blot analysis to assess the specificity of selected bacteriophages. Clones exposing only unique peptides selected from Ph.D-12 and Ph.D-C7C library were used in the experiment. Wild-type bacteriophage M13 that lacks a random peptide insert was used as a negative control. The results are presented in Table 1. Depending on the color density of spots on the nitrocellulose membrane, conventional values were assigned to every spot: symbol ++++ denotes the brightest spots; +—denotes the less intensely colored spots; dash denotes the absence of signal.

**Fig 1:**
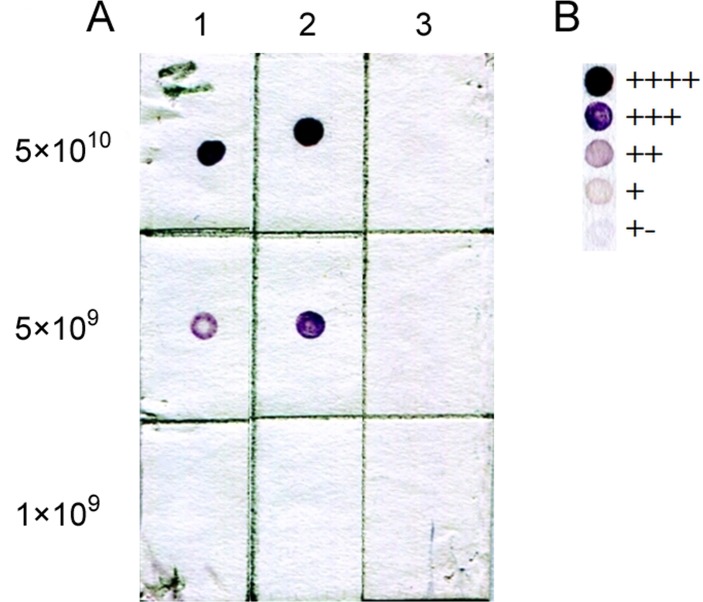
Specific recognition of phage clones selected during biopanning by mAb VRC01. (A) Lane 1, phage clone carrying C4 peptide; lane 2, phage clone carrying C1 peptide; Lane 3, phage clone with no random peptide insert (negative control). Phage titer (in plaque forming units per ml) is labeled to left the dot blot. (B) Spot intensity related to the symbols.

As an example, Fig. 1 demonstrates a scanned image of nitrocellulose membrane with applied serial dilutions of bacteriophage suspensions carrying C4 and C1 peptides and phage that lacks a randomized peptide insertion as a negative control.

### Alignment of peptide sequences exposed on the selected phages and molecular docking

**Fig 2:**
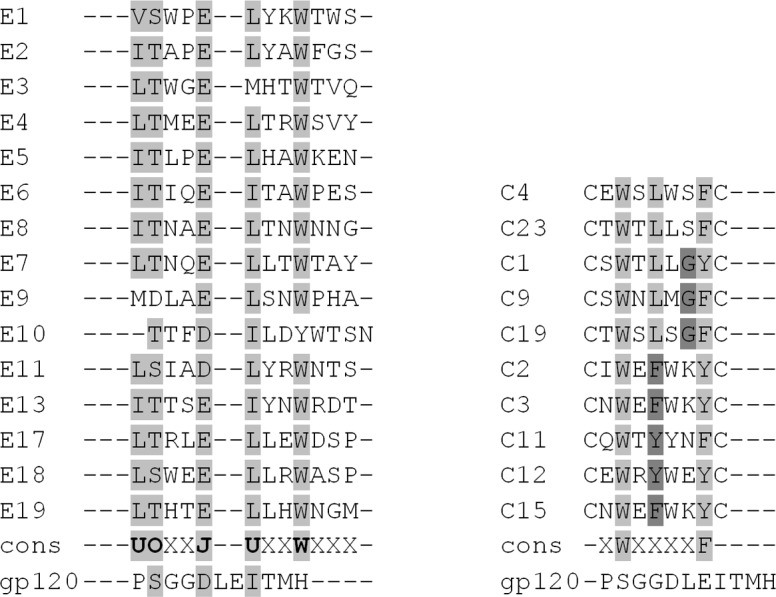
Alignment of the peptides from the phage clones showing the highest binding strength with VRC01 in dot blot with fragment 365–371 of gp120. Amino acid residues that correspond to the consensus sequence are marked by grey.

The majority of phage clones selected from the Ph.D-12 library exposed peptides that have the following structure: UOxxJUxxWxxx, where X is any amino-acid residue, U is a non-aromatic hydrophobic residue (Leu, Ile or Val), O is Ser or Thr, and J is a negatively charged residue (Asp or Glu) (peptide sequences presented in Fig. 2). At the same time, phages isolated from the cysteine constrained 7-mer library had exposed peptides that were similar in 2 amino acid residues, and common motif was xWxxxxF. However, they could be divided into 3 groups consisting of peptides with 3 identical residues (see Fig. 2). Therefore, the resulting common motif may be shown as xWxL/F/YxxF/Y.

To confirm the binding specificity of the peptides with bNAb VRC01, we analyzed the structure of the VRC01–gp120 complex using a model obtained by X-ray crystallography analysis [5]. The V3 and V1/V2 loops of gp120 were removed from this complex since they inhibit crystallization of gp120. The epitope includes the following fragments of gp120: residues 123, 124, and 198, fragments 276–283 (loop D), 365–371 (CD4-binding loop), 427–430, and 455–474 (loop V5). We hypothesized that peptides found by phage display can mimic VRC01 epitope fragments on the surface of gp120.

Using the pdMap tool, we found that the best structural match between 12-mer consensus peptide sequence and epitope was achieved at the gp120 CD4-BS region. A search of the corresponding region of gp120 was initially limited by the residues which are the members of VRC01 epitope. Fig. 2 demonstrates the alignment of gp120 with the peptides from phage clones that were chosen relative to the strongest signals obtained in the dot-blot. Residues Asp368 and Ile371 are among the five key residues that contribute most to the binding of gp120 toVRC01 [24]. The VRC01 epitope does not include any tryptophan, phenylalanine or tyrosine radicals; for this reason, the C-terminal half of the peptide xxWxxx, apparently does not mimic gp120 and contributes to the binding of the peptide to the antibody through another interaction. At the same time, no structural match was found between gp120 CD4-BS region and consensus C7C motif.

### Virus neutralization inhibition assay

**Fig 3:**
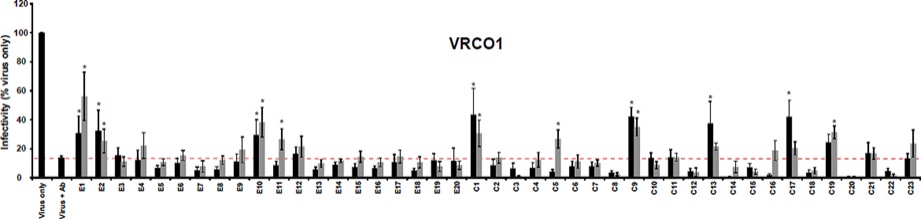
Inhibition of virus-neutralizing activity of monoclonal antibody VRC01 by synthesized peptides. The luciferase activity from virus-only infection was set to 100%. Red dashed line denotes luciferase activity produced by virus with the addition of VRC01 (IC90 = 6.26 μg/ml) without peptides. Black columns correspond to 1:5000 antibody to peptide ratio, grey columns correspond to 1:1000 antibody to peptide ratio). Asterisk indicates statistical significance from the virus/VRC01 control (p ≤ 0.05).

Peptides from selected phage clones were synthesized and checked for their ability to interfere with the neutralization activity of VRC01 using a virus neutralization inhibition assay. The list of synthesized peptides is presented in Table 1. The ratios of antibody:peptide used in the experiment were approximately 1:5000 and 1:1000. It was found that 9 of 44 peptides showed statistically significant rescue of infectivity; however, not all of them demonstrated dose dependence (see Fig. 3). These include four peptides from the 12-mer library (E1, E2, E10, and E11) and five peptides from the C7C library (C1, C5, C9, C13, and C17). The E1 peptide (VSWPELYKWTWS) showed the greatest rescue of HIV-1 infection from VRC01 neutralization.

**Fig 4:**
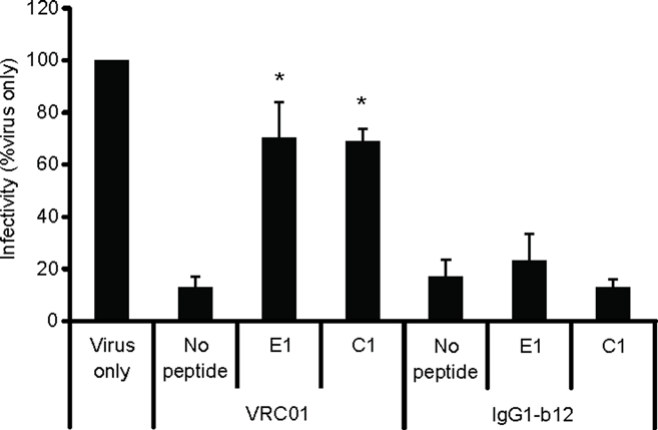
The E1 and C1 peptides specifically relieve neutralization by VRC01. The luciferase activity from virus-only infection was set to 100%. The luciferase activity produced by virus with the addition of VRC01 (IC90 = 6.26 μg/ml) or IgG b12 (IC90 = 12.5 μg/ml) without peptides was used to determine rescue of neutralization. Asterisk indicates statistical significance from the virus/VRC01 or virus/IgG1 b12 controls (p ≤ 0.05).

Additionally, we tested the ability of E1 and C1 peptides to relieve neutralization by mAb IgG1 b12 [22] to verify that these peptides specifically interfere with the neutralization activity of VRC01. Similar to Fig. 3, both E1 and C1 peptides rescued neutralization by VRC01. However, these peptides did not relieve neutralization by IgG b12 (Fig. 4), indicating that the E1 and C1 peptides specifically inhibit VRC01.

We also had attempted to determine if the synthesized peptides could bind to VRC01 in dot blot. However, we had noticed there was significant variability in the affinity of the peptides to the nitrocellulose membrane. Therefore, it was impossible to determine if a lack of signal was due to the peptide not binding to VRC01 or that the peptide had been washed off the membrane during the blotting procedure.

### Model of interaction between peptide fragments SWPEL and VRC01-gp120 complex

**Fig 5:**
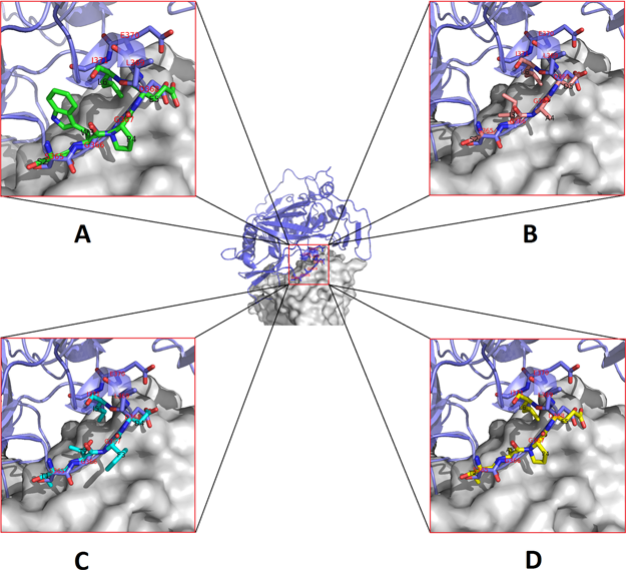
Models of interaction between fragments of the peptides which were able to rescue viral infectivity and VRC01-gp120 complex. Antibody showed as a surface (gray color), secondary structure of gp120 is colored in blue, amino acid residues of SGGDLEI gp120 region is labeled with red, fragments of peptides E1 (SWPEL), E2 (TAPEL), E10 (TTFDI) and E11 (SIADL) are shown as a colored sticks (A, B, C and D, respectively).

To confirm the ability of peptides to bind to bNAb VRC01 at the region of CD4-BS—VRC01 interaction, we have designed four models of fragments of the peptides that were able to rescue viral infectivity. It was found that the fragments of peptides E1 (SWPEL), E2 (TAPEL), E10 (TTFDI), and E11 (SIADL) are able to mimic region 365–371 of gp120 without any steric problems (Fig. 5). Since we did not observe any homology between cysteine-constrained 7-mer peptides and VRC01 epitope, models of interaction between C7C peptides and gp120-VRC01 complex were not constructed.

## Discussion

Phage display is a useful tool for selection of small peptides that mimic immunogenic epitopes of pathogens. Peptide mimics (mimotopes) can be used as a substitute for native antigen for immunization and for eliciting specific antibodies. A number of studies have shown the formation of a specific immune response to mimotopes [25], that were used for development of candidate vaccines against different diseases, such as pneumococcal diseases [26], hepatitis A [27], and cancer [28–30]. Perhaps this approach can be useful for obtaining antigens capable of inducing bNAbs against HIV-1 [13–15, 17, 31].

In our study, the phage display technique was used to select peptides mimicking epitopes that are recognized by bNAb VRC01. For this purpose, affinity selection was carried out with the use of phage libraries Ph.D-12 and Ph.D-C7C (New England BioLabs). In the 12-mer library, peptides are exposed on the surface of M13 bacteriophage as a part of pIII as a linear sequence; in the 7-mer library, peptides are exposed in the shape of a loop fixed by a disulfide bond. Among the phage peptide libraries available for our study, Ph.D-12 has the longest randomized peptides. The longer random peptide sequences could be potentially useful when the target motif consists of discontinuous amino acid residues, and 7-mer peptide is not long enough to cover the target motif. Ph.D-C7C library is recommended for biopanning in the event that the target molecules have complicated conformational structures. Since the exposed peptide is securely fixed, compared to the linear 12-mer sequence, this can provide the higher binding affinity of antibody-peptide interaction.

DNA sequencing of selected bacteriophages and further analysis using MimoDB software enabled us to detect unique peptide sequences selected as the result of biopanning. The binding ability of selected phage clones to bNAb VRC01 were assessed qualitatively using immunoblotting. The color density of spots in dot-blot differed in different phages (Table 1), suggesting differences in the binding affinity of the phage-displayed peptides to VRC01. Fig. 1 shows that VRC01 did not interact with the negative control phage (wild-type bacteriophage M13), but bound to the selected bacteriophages.

We used computer modeling to identify the amino acid residues that are responsible for interaction between peptides and VRC01. Independent analysis of peptide sampling from the 12-mer library and separately from the 7-mer library was conducted. We compared sequences of peptides from the 12-mer library with sequences of gp120 contacting VRC01. We used data concerning the structure of VRC01–gp120 complex obtained by using X-ray crystallography [5]. The best structural correspondence of consensus peptide sequence from the 12-mer library and the VRC01 epitope was achieved in the region of gp120 CD4-BS. It was found that the SWPEL, SIADL, TAPEL and TTFDI amino acid fragments are able to mimic the region 365–371 from gp120 without any steric restrictions (Fig. 5). These results demonstrate that the selected peptides at least partially mimic the CD4-BS loop.

We also carried out alignment of sequences of circular 7-mer peptides from phage clones that demonstrated the strongest signals in dot-blot. After comparing sequences of peptides from the 7-mer library to sequences of gp120 contacting VRC01 and using pdMap software, we did not detect structural similarity between consensus sequence of peptide and epitope recognized by VRC01. However, this is not unprecedented. Previously, it was demonstrated that 12-mer peptides which mimic human mucin 1 protein (MUC1) core peptide had some sequence similarity with MUC1, whereas 7-mer peptides had no homology with MUC1 at the sequence level but were also able to mimic target peptide [32]. Moreover, the immunogenic properties of the constrained 7-mer peptides were even better than the linear 12-mer peptides. This suggests that immunogenic mimicry does not require matching of amino acid residues of peptide mimics and epitopes.

After analyzing the structures of peptides exposed on selected phage clones from both of the libraries, we found that there were no common motifs between peptides from the two libraries that were used. The majority of phage clones selected from the Ph.D-12 library expose peptides of the form UOxxJUxxWxxx, where X is any amino-acid residue, U is a non-aromatic hydrophobic residue (Leu, Ile or Val), O is a Ser or Thr, J is a negatively charged residue (Asp or Glu). Composition of the circular 7-mer peptides is more promiscuous; common motifs have the following structure: xWxL/F/YxxF/Y (Fig. 2). Bacteriophages from both 12-mer and C7C libraries specifically bound to VRC01 in the dot-blot analysis and both 12-mer and C7C peptides were identified in the virus neutralization inhibition assay, indicating that linear and circular peptides bind to VRC01 in different ways.

We also noted that common characteristics of almost every peptide presented on the surface of reactive phage clones in dot-blot is the presence of tryptophan residue in the fourth position from the C-terminus, suggesting that it plays a critical role in peptide binding to the VRC01 antibody. Competitive analysis for mimics binding to VRC01 antibody in virus-neutralization reaction has shown that four peptides from the 12-mer library (E1, E2, E10, and E11) and five peptides from the C7C library (C1, C5, C9, C13, and C17) showed statistically significant rescue of virus infectivity from the neutralizing activity of VRC01 (Fig. 3). These findings showed that the phage-displayed epitopes were able to mimic immunological properties of the native HIV-1epitope recognized by VRC01. However, many synthetic peptides did not interfere with virus neutralization, even though the presence of this sequence resulted in their selection in the biopanning experiments and dot blot analysis. This result can be explained in different ways. First, it is possible that the peptide, as part of the bacteriophage, has more stable structure which results in higher binding affinity to antibody compared to its free state. Second, the synthetic peptides may have a different conformation than phage-displayed peptides, especially the cyclic 7-mer peptides. Peptides exposed as part of bacteriophages from Ph.D-C7C library have cysteines at both termini, which, due to oxidation of the side chain thiols, link together by a disulfide bridge. As a result, the peptides have a circular form. The peptides were not additionally modified and synthesized in linear form. Hence, disulfide bridges could occur between different peptide molecules resulting in multimerization. Third, peptides with specific, but weak, binding to VRC01 may give signal in the dot blot analysis but may be displaced by HIV-1 envelope during infection due to the potentially higher binding affinity of HIV-1 envelope and VRC01. Fourth, linear peptides may aggregate in certain conditions, which is likely since many of them were poorly soluble. This could be also the reason for the absence of dose dependence in competitive neutralization assay (see Fig. 3).

Nevertheless, we have identified peptide mimotopes from both of the linear 12-mer and circular C7C libraries which bind to VRC01 and partially rescue infectivity of HIV-1 NL4–3. One reason for the disparity in mimotope sequences might be that the epitope that is recognized by the mAbs VRC01 is conformational and thus consists of a discontinuous amino acid sequence. The discontinuous amino acids could be in close spatial proximity due to the folding of peptides. It was also demonstrated by many researchers that structural mimicry is not necessarily required for immunogenic mimicry to occur. Moreover, the lack of a common motif could be explained by the fact that the 12-mer linear peptides are flexible, in contrast to cysteine-constrained 7-mer peptides, and bind to the target in other ways.

## Conclusion

Our research findings showed immunological mimicry of a conformational antigenic determinant recognized by monoclonal antibody VRC01 by linear peptides. From the phage display libraries we selected peptides that at least partially mimic a fragment of the CD4-binding loop. Chemically synthesized peptide mimics are able to inhibit neutralizing activity of VRC01. Comparison of mimotopes selected from a peptide library with mAb and the original antigen sequence could lead to a better understanding of the molecular mechanisms that participate in the immune response.

The obtained peptide mimics could be potentially used as a base for computation and construction of artificial immunogens to develop a preventative vaccine against HIV-1. After obtaining peptide mimics recognized by bNAbs of different specificity, it is possible to use them for design of artificial polyepitope B-cell immunogens [15, 33]. However, we believe that the use of these peptides as potential immunogens and the extensive characterization of the immune responses is a large topic and is best addressed in future studies.
